# Skin Rejuvenation Using Autologous Cultured Fibroblast Grafting

**DOI:** 10.7759/cureus.75405

**Published:** 2024-12-09

**Authors:** Yoshie Hirose, Chiharu Fujita, Tomoka Hyoudou, Eisuke Inoue, Hajime Inoue

**Affiliations:** 1 Dermatology, Ginza Yoshie Clinic, Tokyo, JPN; 2 Regenerative Medicine, Laboratory of Cell Applied Technologies Co., Tokyo, JPN; 3 Aesthetic Dermatology, Ginza Yoshie Clinic, Tokyo, JPN; 4 Medical Statistics, Showa University Research Administration Center, Tokyo, JPN; 5 Plastic and Reconstructive Surgery, St. Marianna University School of Medicine, Kawasaki, JPN; 6 Regenerative Medicine, Ginza Yoshie Clinic, Tokyo, JPN

**Keywords:** cultured fibroblast, regenerative medicine, skin rejuvenation, texture, wrinkle

## Abstract

Background: Recently, autologous cultured fibroblast and platelet-rich plasma (PRP) therapies have been attempted for skin rejuvenation. Unlike PRP, grafted fibroblasts not only produce connective tissue but also influence the surrounding environment through a paracrine effect. Fibroblast-derived cytokines interact with and are modulated by neighboring tissue-constituting cells. In this study, we aimed to perform autologous fibroblast therapy and examine its effectiveness for skin rejuvenation through patient and doctor evaluations.

Methods: Eighty-eight patients (5 males and 83 females) were followed up three months after grafting. All cases had a chief complaint of age-related skin atrophy. 1x10^8^ cells of autologous cultured dermal fibroblasts were administered to each face. The patient and doctors evaluated the treatment's effects at one and three months.

Results: One-month post-treatment, 60.3% of the patients rated the treatment as effective, while the doctors evaluated 79.5% as effective. Due to these findings, we assessed the efficacy of the eighty-eight patients at three months by the last observation carried forward (LOCF). The results showed that 75% and 92%, respectively, had effective patient and doctor assessments. The effects of fibroblast treatment were more effective after three months.

Conclusion: Fibroblast grafting was more effective at three months than one month and was extremely effective in improving skin texture, such as sagging, firmness, and wrinkles, without the symptoms of large depressions. There was a natural improvement more than hyaluronic acid injection.

## Introduction

Atrophic skin changes cause wrinkles and saggy faces are often seen as disfigurements. Consequently, these patients are given treatment for severe emotional distress. Invasive procedures, such as fat injections, are not commonly performed; however, non-invasive facial rejuvenation is expected.

Cutaneous atrophy is significantly influenced by ageing and environmental factors. Ageing is an intrinsic phenomenon, whose trajectory differs significantly among individuals, leading to sclerosis and atrophy of the connective tissue, which primarily causes wrinkles and sagging [1]. The skin is constantly exposed to nociceptive stimuli, including environmental elements and ultraviolet rays, which cause skin ageing [2]. Moreover, air pollution and lifestyle factors affect skin ageing [3].

Medical techniques aimed at improving age-related facial appearance involve using hyaluronic acid and collagen as fillers for wrinkles or sagging. Despite the immediate effectiveness of these treatments, their injection can lead to problems, such as heterozygous reactions, allergies, and injection errors. New medical techniques including platelet-rich plasma (PRP) [4] and autologous fibroblast therapies do not carry such risks. Regenerative medicine techniques using PRP and fibroblasts have little risk of human error when administering the filler, and their derivation from autologous tissues helps prevent foreign-body reactions. PRP therapy for anti-ageing on the face has been known for a long time; however, in 1995, Isoragen established a skin dysmorphic therapy using autologous skin tissue-derived fibroblasts [5,6].

The expected effects of PRP and fibroblast grafting therapies on the face are similar. Specifically, they enhance the metabolism of collagen and other connective tissues that decline with age. PRP-derived cytokines activate epithelial cells and surrounding fibroblasts, which stimulate the secretion of MMPs and other connective tissue-degrading enzymes while improving the appearance of skin wrinkles and depression [7]. PRP-derived cytokines stimulate the growth of epithelial cells and surrounding fibroblasts to metabolize old connective tissue and create new tissue by simultaneously promoting collagen synthesis [8].

When using grafted fibroblasts, the cytokines produced exert efficacy by promoting the degradation and synthesis of connective tissue, coupled with effects on surrounding cells [9]. These regenerative medical techniques have the same mechanism of action and are highly anticipated as cell therapies for skin rejuvenation. Fibroblast grafting is expected to maintain long-term functionality. The authors have treated more than 2,000 cases of facial disfigurement with PRP therapy [4] and recently began therapy with autologous cultured fibroblast grafting. In this study, we aimed to perform autologous fibroblast therapy and retrospectively investigate its effectiveness on skin rejuvenation.

## Materials and methods

Patients

The technique of autologous cultured fibroblast grafting was reviewed and approved by the Technical Advisory Committee of the Extraordinarily Certified Committee for Regenerative Medicine of Yukeikai (No: NA8200002), recognized based on the Safety of Regenerative Medicine Act regulated by the Ministry of Health, Labour and Welfare. The ministry gave the following approval numbers to the seven annex clinics, including PB3200155, PB3210049, PB3210122, PB32101125, PB3210143, PB3200146, and PB3220104.

The selection criteria for patients undergoing autologous cultured fibroblast grafting were as follows: patients who were asked to undergo autologous fibroblast grafting therapy and had skin or face disfigurement associated with age. The exclusion criteria are as follows: if there is a disease for which therapy should take precedence over this treatment, a malignant disease (e.g., cancer), a case of disagreement on informed consent, or a case of non-compliance with this therapy.

The treatment was performed after obtaining verbal and written consent from the patients. 88 patients (83 females and five males; age 54.1±11.1 years) were included in the study, with a follow-up period of either one or three months, or both, after surgery between January 1, 2021, and December 31, 2023. The results of these patients were evaluated retrospectively.

Fibroblast culture

Four skin samples (4 mm in diameter) were obtained from the site requested by the patient, and the physician diagnosed them by using a biopsy punch. The skin obtained was sectioned into small pieces under good sanitary conditions, placed at the bottom of a culture dish, and fixed with a cover glass. The Dulbecco-modified eagle medium containing 10% fetal bovine serum was added, and the cells were cultured at 37°C in a 5% carbon dioxide environment. The tissue pieces were stored for approximately 10 days until fibroblast migration was observed. Following confirmation of cell migration, the fibroblasts were cultured in the same medium until confluent and passaged using the standard procedure. After centrifugation at 1200 rpm for 5 min, the cell sediment was washed twice with phosphate-buffered saline (PBS) without Calcium and suspended with saline for injection at a defined cell number (1-2×10^7^ cells/mL) using injectable saline.

Grafting

Following surface or block anaesthesia of the grafting area, a dose of approximately 1×10^8^ cells/total suspended in 5-10 mL saline was administered to the entire face using a 31G×4 mm injection needle, with a slightly larger volume on the major complaint region. After cell grafting, the face was cooled on ice for approximately 30 minutes to relieve pain after the local anaesthetic wore off.

Post-grafted management

Following fibroblast grafting, patients were limited to face washing on that day only and resumed other activities the following day.

Statistical analysis

The results were independently evaluated by patients and physicians on a 5-point scale (1=unsatisfactory, 2=slightly unsatisfactory, 3=unchanged or unclear, 4=slightly satisfied, and 5=satisfied) one and three months after the procedure, according to the method reported by Kamakura et al. [10]. In all cases, a rating of three or lower was considered invalid, and four or higher was considered effective. The fibroblast grafting therapy was considered effective if ≥50% of patients were satisfied (rated four or higher) at follow-up. Comparisons were performed using the Binomial test, with a significance level of 0.05. Missing data at three months were imputed with appropriate values depending on the reason. In cases where patients failed to present for a follow-up due to forgetfulness or a perceived good outcome, values recorded at one month were used as those observed at three months. Cases lost to follow-up and needing follow-up treatment at three months received the lowest observed scores for analyses. Changes in patient and physician assessments were examined using the Mc Nemar test with a significance level of 0.05.

## Results

Typical patient findings

**Figure 1:**
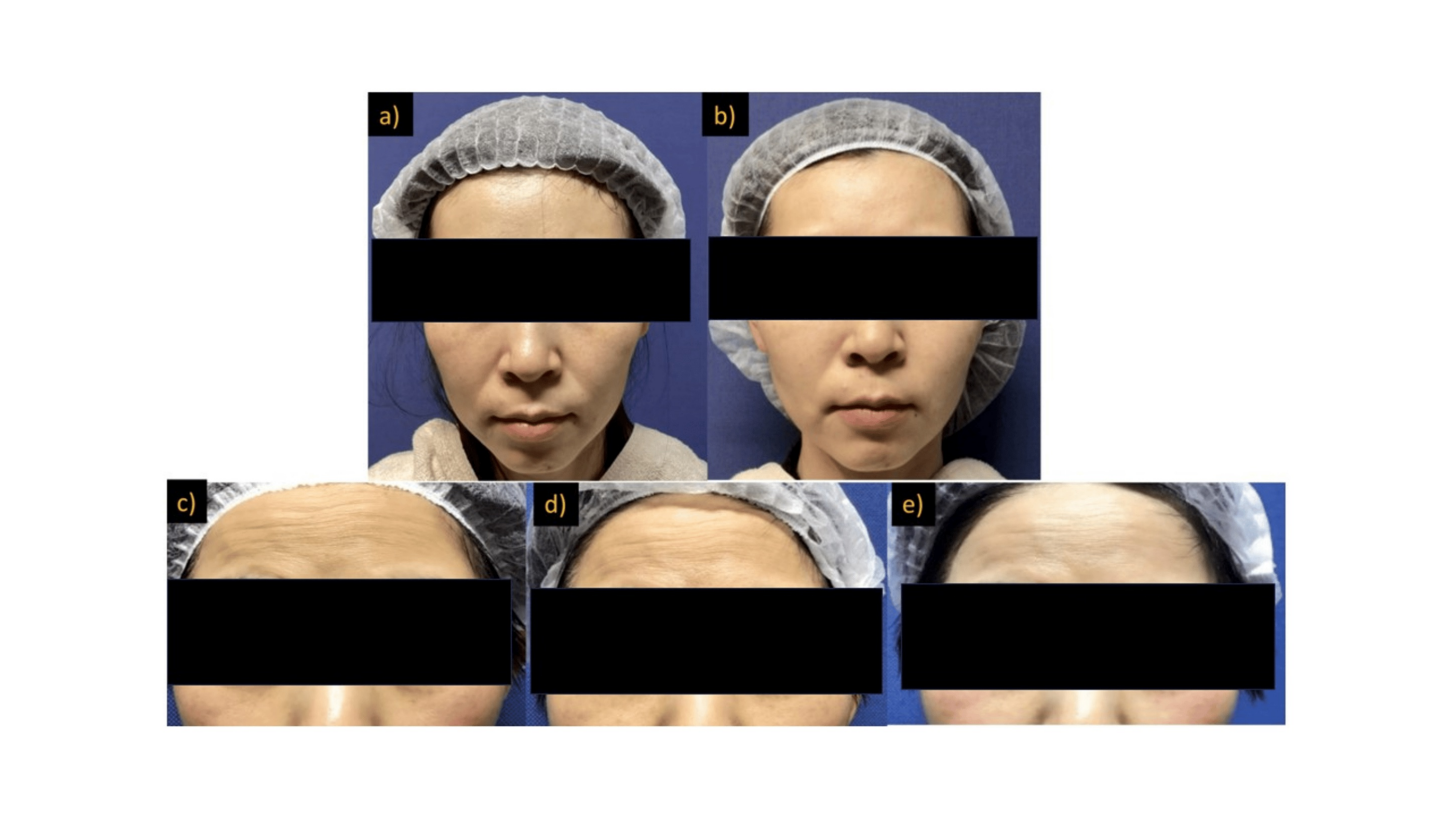
Typical photograph before/after cultured fibroblast therapy. The patients were (a, b) female aged 40 years old (c, d, e)  female aged 50 years old.  Fibroblasts were injected according to the grafting procedure described in Materials and Methods. a) before treatment, b) three months after treatment, c) before treatment, d) one month after treatment, e) three months after treatment. The grooves of the nasolabial line had already changed effectively three-months postoperatively (figures a, b). There was a marked improvement in skin texture and elasticity compared to those before application. In the lower patient's case (Figures C, d, e), the improvement in wrinkles on the forehead (fine lines) was also observed to be more effective than that of the 40-year-old patient (upper).

All patients who requested therapy had the following primary complaints: low skin firmness, texture, wrinkles, and sagging. There have been a few requests for therapy due to skin depression. Figure 1 shows two typical patients after fibroblast application. There was a marked improvement in firmness, elasticity, and skin texture compared with those before the application. Improvement in the crepe wrinkles were observed.

All patients who rated the treatment as effective reported improvements in skin texture and firmness (elasticity and sagging). Similarly, physicians who found the treatment effective noted improvements, especially in fine wrinkles.

Post-treatment evaluation

**Figure 2:**
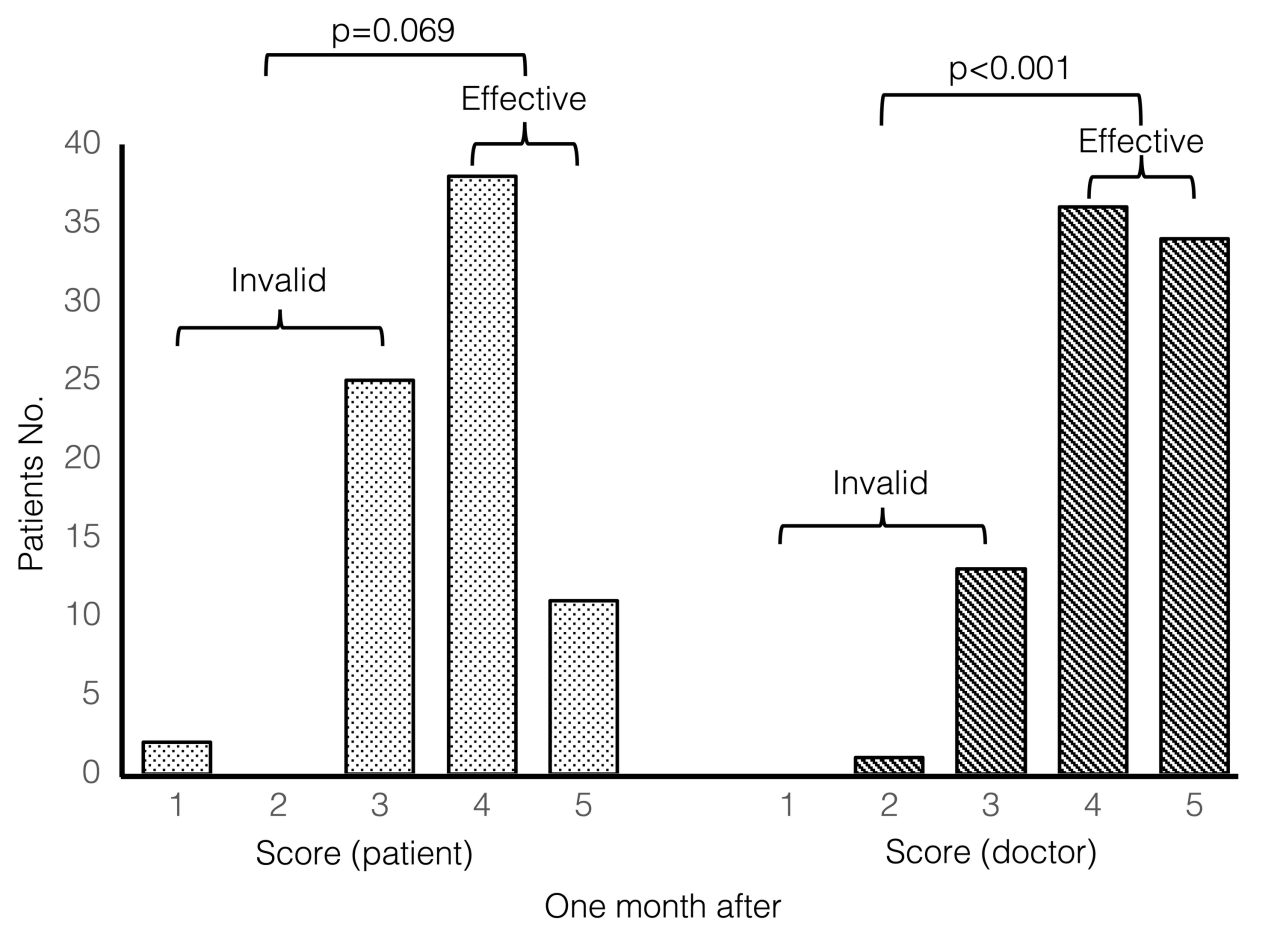
Patient and physician outcome evaluation scores obtained a month post-treatment (n=84) Patient assessment: Invalid (<3); 31 cases (35.2%), Effective (>4); 53 cases (60.3%). Physician assessment: Invalid (<3); 14 cases (15.9%), Effective (>4); 70 cases (79.5%). A binomial test was used for statistical significance testing, with a significance level of 0.05.

Out of the 88 patients who received fibroblast therapy, 84 were evaluated after a month, and the patient and physician ratings were as follows. A total of 70 patients (79.5%, 95% confidence interval: 69.6-87.4%) had effective physician-based assessments, and 53 patients (79.5%, 95% confidence interval: 49.2-70.5%) had effective self-assessments (Figure 2). Physician evaluations were effective statistically significantly (p<0.001) but patient evaluations were not significant (p=0.069).

Among the 56 patients who were evaluated after three months, the patient and physician ratings were as follows. Overall, 32 patients could not be evaluated due to forgetting to come to the hospital rather than being lost to follow-up. The efficacy evaluation of these patients at one month revealed 13 and 19 patients (40.6% and 59.4%), respectively, with an invalid and valid rating. In the physician evaluation, four and 28 cases (12.5% and 87.5%) gave an invalid and effective evaluation, respectively. Therefore, it could not be assumed that patients with invalid evaluations at one month abandoned the consultation after three months.

**Figure 3:**
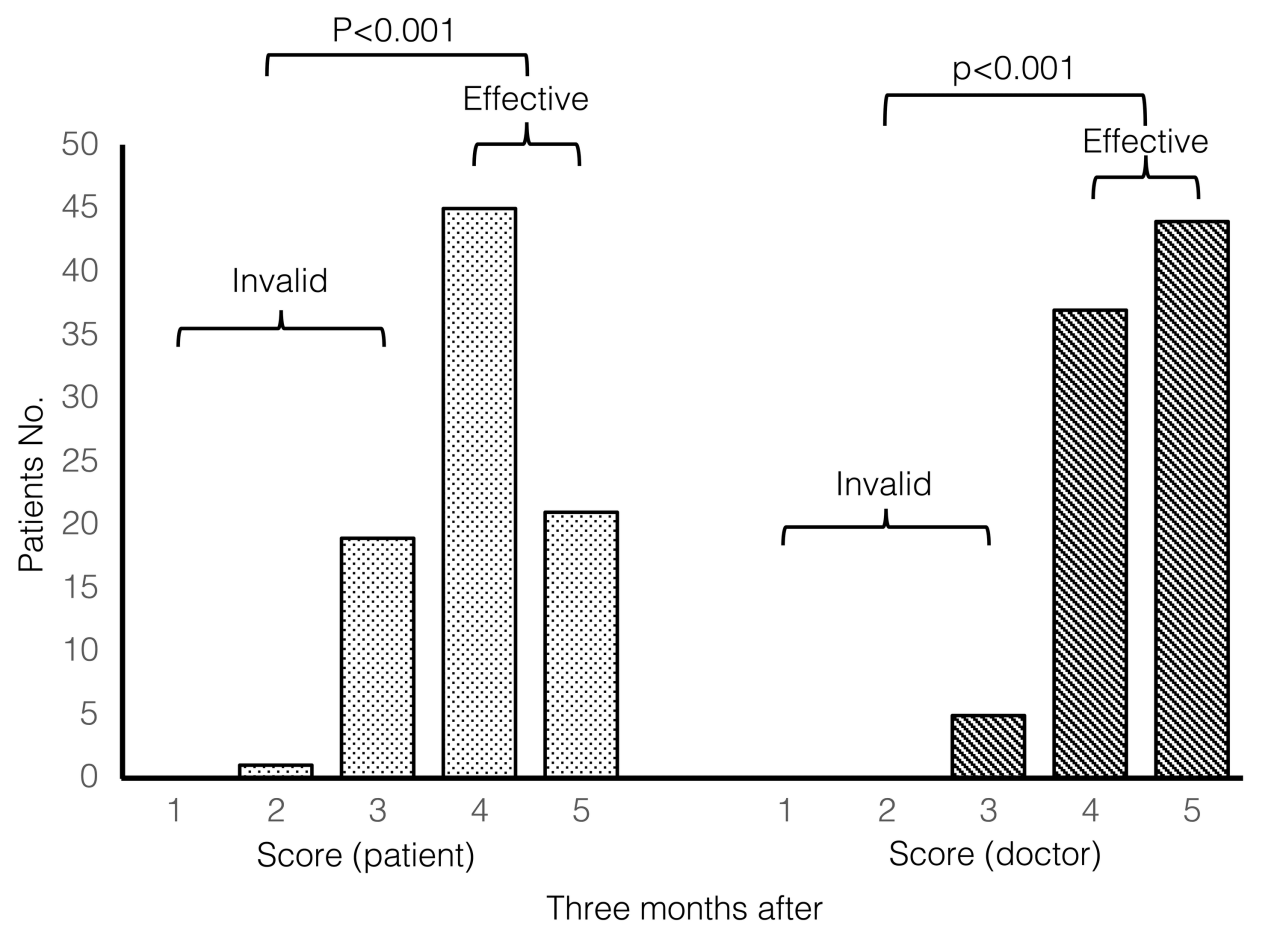
Patient and physician improvement scores at three months post-treatment (n=88) Patient assessment Patient assessment: Invalid (<3); 22 cases (25.0%), Effective (>4); 66 cases (75.0%). Physician assessment: Invalid (<3); 7 cases (8.0%), Effective (>4); 81 cases (92.0%). A binomial test was used for statistical significance testing, with a significance level of 0.05.

As the missing data were independent of the outcome, we imputed the missing three months' data using the values obtained in one month. With the imputed dataset, the results showed that 66 patients (75.0%, 95% confidence interval: 64.6-83.6%) and 81 patients (92.0%, 95% confidence interval: 84.3-96.7%) had valid patient and physician assessments, respectively (Figure 3). Patient evaluations were effective and statistically significant (p<0.001). Physician evaluations were also statistically significant (p<0.001).

**Figure 4:**
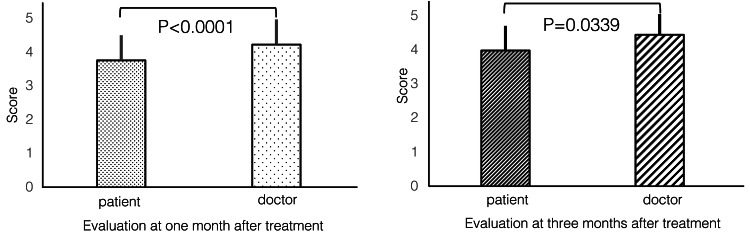
Patient and physician score differences at evaluations points. The results showed that physician evaluation was significantly higher than patient evaluation at both the one (p<0.0001) and three months (p=0.0339) evaluations. McNemar’s test was used for statistical significance testing, with a significance level of 0.05.

Discrepancies between the physicians’ and patients’ efficacy ratings were evaluated. The results showed that physician evaluations were significantly higher than patient evaluations at one and three months (p<0.0001 and p=0.0339) (Figure 4).

**Figure 5:**
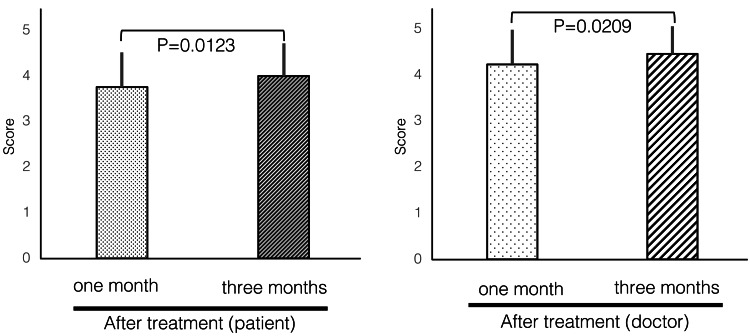
Change of improvement scores after treatment The effects of fibroblast therapy were examined at one and three months after administration. Both patients (p=0.0123) and physicians (p=0.0209) evaluated fibroblast therapy as more effective at one and three months after treatment. McNemar’s test was used for statistical significance testing, with a significance level of 0.05.

Furthermore, the effects of fibroblast administration at one and three months were examined. Both patients (p=0.0123) and physicians (p=0.0209) evaluated fibroblast treatment as effective, observing higher efficacy at three months compared to that at one month (Figure 5).

## Discussion

All patients treated in this study had primary complaints of age-related loss of firmness, texture, wrinkles, and sagging. In contrast, fewer patients complained primarily of depressed nasolabial fold or marionette lines. This may be because PRP or fibroblasts were administered to patients who were informed at the time of consent that immediate effects of the filler could not be expected. To solve this problem, it is to be injected into the suspended cultured cells or PRP in hyaluronic acid [11,12]. However, hyaluronic acid is used as an inhibitor of tissue adhesion [13], and it is uncertain if the applied fibroblasts are attached to the grafted site. These combined therapies should be administered based on the physiological and pharmacological evidence and mechanism.

Fillers such as hyaluronic acid are effective in the treatment of skin disfigurement with depressions; however, the physiological environment of the dermis around the injection site greatly deviates from the anatomical state [14]. The result is determined by the injection technique; however, the injection site often loses its natural appearance. Fat injection is an effective therapy; however, fat collection is an invasive procedure, and internal bleeding may occur after injection [15]. Treatments such as phototherapy are known to be effective [16].

Mechanism of action

Recently, PRP therapy has been widely used for skin disfigurement. However, it is used as a regenerative medicine technology in the fields of orthopaedics [17] and dentistry [18]. Anti-ageing therapy using PRP is not immediately effective but is highly recommended for the natural improvement of wrinkles [4,19]. The mechanism of action may involve the administered platelets signaling the host to initiate wound healing. Tissue regeneration during the wound healing process fills the atrophied connective tissue and promotes epidermal turnover, thereby improving sagging and dullness.

Homology of the mechanism of action

Laser therapy stimulates tissue regeneration by initiating wound healing through mild damage to the skin and may have the same mechanism as PRP [16]. During wound healing, fibroblasts created in the dermis are involved in the production of connective tissues such as proteoglycans and collagen [20]. Fibroblast proliferation and connective tissue production are influenced by growth factors from various cells (tissue engineering) [21], while the cytokines and growth factors produced by fibroblasts affect the metabolism of surrounding skin tissues, including vascular endothelial cells and keratinocytes. This interaction is a paracrine effect and may be important for skin rejuvenation [22]. Therefore, the administration of fibroblasts affects the proliferation of connective tissue and acts on the surrounding skin cells (especially existing fibroblasts), which may affect connective tissue metabolism and epithelial cell turnover.

Standardization of autologous cell therapy

Fibroblasts are cell-derived devices used for skin rejuvenation, similar to PRP. Although their effectiveness is widely recognized, cell products using components derived from autologous tissues have large individual differences, unlike the use of drugs or allogeneic cell products. This phenomenon is a major cause of loss of trust in the effectiveness of regenerative medicine. Popescu et al. have reported that it is difficult to evaluate platelet-rich plasma therapy's quality control and efficacy unless the blood collection method, dosage and frequency, and administration method are standardized [23]. They also report that standardization is desirable.

Fibroblasts are similar, but unlike PRP, treatment using fibroblasts requires culture and grow fibroblasts. In other words, since each patient's cells are maintained in the same environment until they are used in therapy, it is impossible to regulate individual differences in cell function, but if the culture medium and cell recovery method are determined, the state of the cells may be standardized to a certain extent. On the other hand, if the therapeutic method is standardized using administration techniques for cosmetic medicine such as hyaluronic acid, it may be thought that the variation due to the technique can be greatly reduced. Such standardization may make it possible to evaluate the effectiveness objectively to a certain extent.

PRP and fibroblast-based therapy is the oldest representative cosmetic regenerative medical technique. Recently, adipose tissue-derived stromal cell (stem cell) therapy has also been attempted [24]. However, the differences among these three therapies in terms of cosmetic purposes are unclear. Furthermore, there are no criteria for cell selection based on the mechanism of action. Consequently, approaches are often selected based on patient preference and physician judgment. In the future, an objective list of indications, approaches, and evaluation methods may be useful as the patient population increases and available techniques improve.

Limitations of this study

Outcome evaluations were higher among physicians than among patients at both follow-up points. This was assumed because physicians made their evaluations based on the balance of the entire face, whereas patients focused on the area of greatest concern rather than the overall change, possibly due to the two-dimensional evaluation using a mirror. Quantifying anti-ageing treatment is challenging. Some authors have attempted objective evaluations using devices that measure wrinkle depth and skin elasticity. However, these measurements poorly correlate with patient evaluations. As a result, subjective patient-based outcomes tend to receive greater weight than objective measurement-based outcomes. We should explain to patients detailed information on evidence-based procedures and must ask them to make calm decisions. These therapies cannot be tried with RCT. We must discuss the obtained results on the assumption that they include the patient's expectations as well as the physician's wishes.

## Conclusions

Fibroblast grafting was more effective at three months than one month and was extremely effective in improving skin texture, such as sagging, firmness, and wrinkles, without the symptom of large depressions. There was a natural improvement more than hyaluronic acid injection.We further conclude that skin rejuvenation using autologous cultured fibroblasts grafting is not an immediate effect, but it is effective in the long term and is expected to produce a natural finish. However, its effect on deep depressions (wrinkles) is limited. If its therapy for deep wrinkles is objective, the standard technique using hyaluronic acid may be used with fibroblast or PRP therapy. In the future, it will also be necessary to logically differentiate between this method and regenerative medicine techniques such as PRP at rejuvenation therapy.
